# A Targeted Next-Generation Sequencing Panel to Genotype Gliomas

**DOI:** 10.3390/life12070956

**Published:** 2022-06-24

**Authors:** Maria Guarnaccia, Laura Guarnaccia, Valentina La Cognata, Stefania Elena Navone, Rolando Campanella, Antonella Ampollini, Marco Locatelli, Monica Miozzo, Giovanni Marfia, Sebastiano Cavallaro

**Affiliations:** 1 Institute for Biomedical Research and Innovation, National Research Council, Via P. Gaifami 18, 95126 Catania, Italy; maria.guarnaccia@cnr.it (M.G.); valentina.lacognata@cnr.it (V.L.C.); 2 Laboratory of Experimental Neurosurgery and Cell Therapy, Neurosurgery Unit, Fondazione IRCCS Ca’ Granda Ospedale Maggiore Policlinico, Via Francesco Sforza 35, 20122 Milan, Italy; laura.guarnaccia@policlinico.mi.it (L.G.); stefania.navone@policlinico.mi.it (S.E.N.); campanella.rolando@gmail.com (R.C.); antonella.ampollini@policlinico.mi.it (A.A.); marco.locatelli@policlinico.mi.it (M.L.); giovanni.marfia@policlinico.mi.it (G.M.); 3 Department of Clinical Sciences and Community Health, University of Milan, Via Festa del Perdono 7, 20122 Milan, Italy; 4 “Aldo Ravelli” Research Center, Via Antonio di Rudinì 8, 20142 Milan, Italy; 5 Department of Medical-Surgical Physiopathology and Transplantation, University of Milan, Via Francesco Sforza 35, 20122 Milan, Italy; 6 Department of Health Sciences, University of Milan, 20122 Milan, Italy; monica.miozzo@unimi.it; 7 Unit of Medical Genetics, ASST Santi Paolo e Carlo, 20142 Milan, Italy; 8 Clinical Pathology Unit, Aerospace Medicine Institute “A. Mosso”, Italian Air Force, Viale dell’Aviazione 1, 20138 Milan, Italy

**Keywords:** glioblastoma, glioma, targeted sequencing, precision medicine, genomics

## Abstract

Gliomas account for the majority of primary brain tumors. Glioblastoma is the most common and malignant type. Based on their extreme molecular heterogeneity, molecular markers can be used to classify gliomas and stratify patients into diagnostic, prognostic, and therapeutic clusters. In this work, we developed and validated a targeted next-generation sequencing (NGS) approach to analyze variants or chromosomal aberrations correlated with tumorigenesis and response to treatment in gliomas. Our targeted NGS analysis covered 13 glioma-related genes (*ACVR1*, *ATRX*, *BRAF*, *CDKN2A*, *EGFR*, *H3F3A*, *HIST1H3B*, *HIST1H3C*, *IDH1*, *IDH2*, *P53*, *PDGFRA*, *PTEN*), a 125 bp region of the *TERT* promoter, and 54 single nucleotide polymorphisms (SNPs) along chromosomes 1 and 19 for reliable assessment of their copy number alterations (CNAs). Our targeted NGS approach provided a portrait of gliomas’ molecular heterogeneity with high accuracy, specificity, and sensitivity in a single workflow, enabling the detection of variants associated with unfavorable outcomes, disease progression, and drug resistance. These preliminary results support its use in routine diagnostic neuropathology.

## 1. Introduction

Human gliomas are primary tumors of the central nervous system (CNS) that include different subtypes for their histopathology features and degree of malignancy. Glioblastoma is the most aggressive, invasive, and undifferentiated glioma with a median survival of 15 months [1]. In 90% of cases, it is a primary glioblastoma that arises without previous disease, while secondary glioblastoma is rare and develops from a lower grade astrocytoma [2,3]. Hardly histologically distinguishable, primary and secondary glioblastomas present different clinical features and affect patient groups of different ages and sexes. For example, secondary glioblastomas manifest predominantly in younger patients (median age of 45 years), with a better prognosis compared to primary glioblastomas (median age of 60 years) [4,5]. Based on gene expression, the Cancer Genome Atlas (TCGA) classifies glioblastomas into proneural, neural, mesenchymal, and classical subtypes [6,7]. Secondary glioblastomas are essentially proneural, whereas primary glioblastomas belong to any of the indicated subtypes [8]. More specifically, in the oncogenesis of glioblastomas, biologically relevant alterations in three essential signaling pathways have been reported: RTK/RAS/PI3K (88%), p53 (87%), and retinoblastoma protein (78%) [9]. Other pathways include cell cycle, DNA damage responses, cell death and differentiation, neovascularization, and additional key players, such as Kras, G2M checkpoint, and Wnt [10,11].

**Figure 1:**
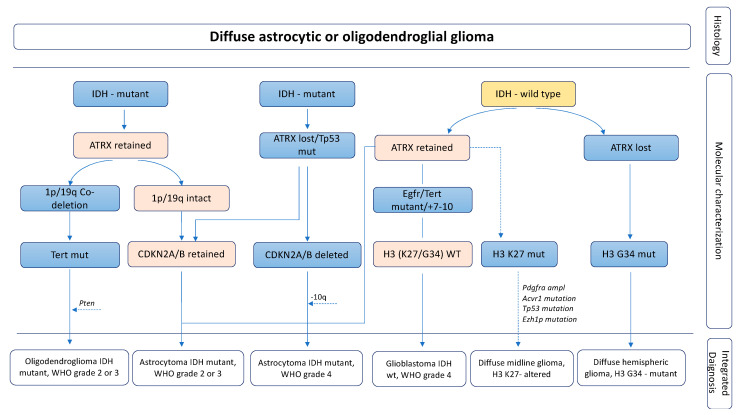
Integrated histomolecular classification of diffuse gliomas according to the 2021 WHO Classification of Tumors of the Central Nervous System complemented by the Consortium to Inform Molecular and Practical Approaches to CNS Tumor Taxonomy (cIMPACT-NOW).

The fifth edition of the WHO Classification of Tumors of the Central Nervous System (WHO CNS5), published in 2021, renamed glioblastoma as “glioblastoma, *IDH*-wild-type” (from now on referred to as GBM) and suggested supplementary guidelines for the histological classification of gliomas, indicating a spectrum of diagnostic, prognostic and/or predictive molecular biomarkers (Figure 1) [12]. With regard to genetic alterations, GBM is typically isocitrate dehydrogenase (*IDH*)-wild-type and has epidermal growth factor receptor (*EGFR*) amplification or mutations, phosphatase and tensin homolog (*PTEN*) deletion or mutations, and loss of heterozygosity on 10q [2]. Additional glioma subtypes may have recurrent point mutations in *IDH1* and *IDH2*, platelet-derived growth factor receptor (*PDGFR*), cyclin-dependent kinase inhibitor 2A (*CDKN2A*) deletion, and tumor protein P53 (*TP53*) [13]. According to the WHO CNS5 classification, complemented by the Consortium to Inform Molecular and Practical Approaches to CNS Tumor Taxonomy (cIMPACT-NOW), *IDH*-mutated gliomas can be further subdivided into astrocytomas (grade 2, 3, or 4) and oligodendrogliomas with 1p/19q codeletion status (grade 2 or 3) [12,14,15] (Figure 1). Additional genetic markers are considered potentially relevant for tumor classification, such as the concurrent gain of whole chromosome 7 and the loss of whole chromosome 10, and mutations in the telomerase reverse transcriptase (*TERT*) promoter that are strongly correlated with glioblastoma *IDH*-wild-type, while mutations of B-Raf proto-oncogene (*BRAF*) and H3.3 histone A (*H3F3A*) associated with alterations of X linked intellectual disability (*ATRX*) and tumor protein P53 (*TP53*) are common in pediatric GBM [16,17,18]. Driver mutations (K27M and G34R/V) in H3 clustered histone 2 (*HIST1H3B*) are linked to pediatric and young high grade GBM with poor outcome [19].

The combinatory use of both histopathological and molecular features by the WHO CNS5 is nowadays mandatory for the diagnostic classification of gliomas [12,20]. Molecular testing includes the evaluation of markers with predictive and/or prognostic value, such as the O6-methylguanine (O6-MeG)-DNA methyltransferase (*MGMT*) promoter methylation, the mutational status of *IDH1/2*, and the presence of chromosomes 1p/19q codeletion [21]. Typically, Karnofsky performance status (KPS) > 70, age at diagnosis < 50 years, *MGMT* methylation (>9%), gross/subtotal resection (>90%), and location of tumor in a non-eloquent area of the brain represent the most favorable prognostic indicators [22]. Loss of heterozygosity (LOH) of 1p/19q is another prognostic indicator of better prognosis, while *MGMT* promoter methylation status is the strongest prognostic factor for GBM outcome and a strong predictor of response to alkylating chemotherapy such as temozolomide (TMZ) [23]. Therefore, treatment strategies in patients with gliomas are based on tissue diagnosis and evaluation of molecular markers. The current standard protocol for treatment of all newly diagnosed gliomas is gross total resection, when feasible, followed by radio- and chemotherapy with TMZ. In addition, therapies with alkylating agents of the nitrosourea class, such as lomustine, carmustine, nimustine, or fotemustine have been used but without effective benefits [24]. Preclinical studies suggest that the combination of TMZ with bevacizumab, an anti-VEGF antibody approved in the United States, Canada, Switzerland, and several other countries outside the European Union for the treatment of recurrent GBM, temporally improves clinical outcomes and quality of life but does not impact overall survival (OS) [24]. However, despite current therapeutic treatments, GBM often inexorably recurs in the same location or in another area of the brain about 14–15 months after neurosurgery.

While enormous advances in the field of both molecular biology and genetics have elucidated the complex etiopathogenesis of gliomas and identified new potential treatment options that target molecular receptors and signaling pathways, only limited progress has been achieved in clinical practice to help the transition towards molecular medicine and personalized approaches. Several molecular signatures can influence tumor biology and impact clinical outcomes, offering the possibility to stratify patients into diagnostic, prognostic, and therapeutic clusters [4]. To this end, the application of different high-throughput technologies, such as NGS, may aid to enable the comprehensive detection of relevant genetic alterations for gliomas according to the WHO classification, as well as to identify those associated with drug resistance [25,26]. However, due to its complexity and challenging bioinformatics pipeline, NGS has not yet entered into routine diagnostic practice, fully replacing established low-resolution techniques [27].

In this article, we describe the development and validation of a custom targeted NGS approach for the analysis of 13 glioma-related genes (*ACVR1*, *ATRX*, *BRAF*, *CDKN2A*, *EGFR*, *H3F3A*, *HIST1H3B*, *HIST1H3C*, *IDH1*, *IDH2*, *P53*, *PDGFRA*, *PTEN*), a 125 bp region of the *TERT* promoter (*TERT*p), and 54 single nucleotide polymorphisms (SNPs) located along chromosomes 1 and 19 for the reliable assessment of their copy number alterations (CNAs).

## 2. Materials and Methods

### 2.1. Sample Collection

Tissue samples from patients who underwent surgical resection of newly diagnosed gliomas (n = 34) were collected at the Neurosurgery Unit of Fondazione IRCCS Ca’ Granda Ospedale Maggiore Policlinico and processed at the Laboratory of Experimental Neurosurgery and Cellular Therapy of the same Institution. Histological subtypes were classified on the basis of morphologic and immunohistochemical characteristics according to the WHO 2016 classification criteria [28]. The clinical and molecular characteristics of the patients (described below in Section 3) were extracted from medical records and included sex, age, *MGMT* methylation, *IDH1*/2 mutation status, 1p/19q LOH, and Ki-67 (MIB-1) labeling index. Methylation of *MGMT* promoter (GRCh37/hg19 chr10: 131.265.471-131.265.581) was assessed by pyrosequencing with the “Pyromark Q96 ID System”. The assessment of 1p/19q LOH, hotspot mutations in *IDH1* (c.394C > T p.R132C, c.394C > G p.R132G, c.394C > A p.R132S, c.395G > A p.R132H, c.395G > T p.R132L, c.395G > C p.R132P), *IDH2* (c.514A > C p.R172R, c.514A > G p.R172G, c.514A > T p.R172W, c.515G > A p.R172K, c.515G > C p.R172T, c.515G > T p.R172M, c.516G > A p.R172R, c.516G > C p.R172S, c.516G > T p.R172S), and *TERT*p mutations (G228A and G250A) was performed using the MassARRAY Analyzer 4 system (Agena Bioscience, San Diego, CA, USA), based on MALDI-TOF mass spectrometry. Tumor proliferation was assessed by Ki-67 (MIB-1) staining using immunohistochemistry, in a range between 15% and 80%. Immunohistochemistry was performed using the BenchMark Ultra automatic system (Ventana Medical Systems) and was reviewed by two independent pathologists.

The Institutional Review Board approved the protocol (IRB#1670/2015), and all patients provided informed consent. All methods were performed in accordance with ethical regulations.

Notably, all patients followed Stupp therapeutic protocol consisting of maximal safe surgical resection, followed by radiotherapy (60 Gy) with concomitant daily TMZ chemotherapy, and then maintenance treatment with TMZ for 6 to 12 months. The approved conventional schedule was a daily dose of 120 to 200 mg per square meter of body-surface area for 5 days of every 28-day cycle [29].

### 2.2. DNA Extraction and Dosage

DNA from FFPE samples was extracted with a DNeasy Blood and Tissue Kits Isolation kit (Qiagen, Germantown, MD, USA), quantified by a NanoDrop ND-3000 spectrophotometer, and assessed for quality by microcapillary electrophoresis on a 2100 Bioanalyzer (Agilent Technologies, Palo Alto, CA, USA). The DNA yield was quantified using a TaqMan RNase P Detection Reagent Kit (Thermo Fisher Scientific, Waltham, MA, USA) on Real Time PCR AriaDx (Agilent Technologies, Santa Clara, CA, USA).

### 2.3. NGS Panel Design

A custom “on-demand” NGS panel was designed using custom IonAmpliSeq Designer software (https://ampliseq.com, accessed on 3 May 2020, Thermo Fisher Scientific, Waltham, MA, USA). The panel included 441 amplicons with a size range of 125–175 bp distributed across two primer pools (Pool_1: 221 amplicons, Pool_2: 220 amplicons) covering 47.22 kb and targeting the whole coding region of 13 glioma-related genes (*ACVR1*, *ATRX*, *BRAF*, *CDKN2A*, *EGFR*, *H3F3A*, *HIST1H3B*, *HIST1H3C*, *IDH1*, *IDH2*, *P53*, *PDGFRA*, *PTEN*), a 125 bp region of the *TERT* promoter, and 54 SNPs located along chromosomes 1 and 19 (Table 1). The in silico coverage was 99.11%. The complete design of the panel is available in Supplementary Material S1 (Tables S1 and S2). Targeted genes and SNPs are detailed below and were selected according to the WHO diagnostic requirements for molecular testing of gliomas [12], a previous gene-set used for targeted strategies [30], and their high mutation frequency in gliomas.

### 2.4. Library Preparation

Automated library preparation was carried out with 0.67 ng of input DNA using an Ion AmpliSeq Kit for Chef DL8 (DNA to Library, 8 samples/run) on the Ion Chef System (Thermo Fisher Scientific, Waltham, MA, USA). Library quality and molarity were assessed by using an Ion Library TaqMan Quantitation Kit (Thermo Fisher Scientific, Waltham, MA, USA) on the Aria Dx Real-Time PCR System (Agilent Technologies, Santa Clara, CA, USA). Serial dilutions of the *E. coli* DH10B control library (Thermo Fisher Scientific, Waltham, MA, USA) were prepared and run in triplicate to generate a standard curve. Molar concentration of libraries was determined by using the Delta R baseline-corrected raw fluorescence calculated with Aria DX Real-Time PCR Software (Agilent Technologies, Santa Clara, CA, USA). Barcoded libraries were super-pooled in equimolar concentrations using the strategies suggested for combining libraries prepared with different panels for equal coverage to obtain final molarity of 60 pM each.

### 2.5. Chip Loading and Sequencing

Loading of the Ion 540 chips was carried out using an Ion 540 Kit-Chef (Thermo Fisher Scientific, Waltham, MA, USA) following manufacturer instructions. High throughput sequencing runs were carried out on an Ion Gene Studio S5 system (Thermo Fisher Scientific, Waltham, MA, USA). Run planned in the S5 Torrent Suite (v. 5.12.2) had the following parameters: analysis parameters, default; reference library, hg19; target regions, IAD199863.bed file; read length, 30X; flows, 550; base calibration mode, default. Plugins used were Coverage Analysis, Ion Reporter Uploader, and Variant Caller (somatic_low_stringency_540).

### 2.6. Analytical Specificity and Sensitivity

To evaluate the performance of variant calls in detecting the true genotype for each sample, we estimated diagnostic accuracy, sensitivity, and specificity of the test by comparing our results with the previous data obtained using different technology (MassARRAY) [31]. True positives (TPs) were defined as correctly genotyped samples whose alternative variants were detected by both our filtering pipeline as well as from MassARRAY collected data. True negatives (TNs) were samples known to be wild-type (wt) from MassARRAY data. False positives (FPs) and false negatives (FNs) were considered as samples carrying variants detected by our pipeline but not from expected data and vice versa, respectively. Accuracy was calculated as follows: (TP + TN)/(TP + FP + TN + FN); sensitivity was calculated as follows: TP/(TP + FN); specificity was calculated as follows: TN/(TN + FP).

The accuracy of NGS-based analysis for somatic genomic variants is also based on the correct detection of mutations at low variant allele frequencies (VAF) [32]. In the NGS method, this parameter is assessed by measuring the limit of detection (LOD). Different factors such as nucleic acid input, processing methods, or sequencing depth can influence LOD [33]. Moreover, in cancer screening, tissue heterogeneity caused by the co-existence of multiple mutations in tumor cells or the occurrence of multiple clones in the same tumor is another factor that can affect this metric [34]. To assess the accuracy of our method in the detection of somatic mutations at any VAF in a tumor sample, we measured the LOD using diluted DNA samples. Pre-validated genomic DNA samples were used as reference material and contained different types of mutations. The mix of known reference DNA dilutions with wild-type DNA was used as template, and a theoretical LOD ranging between 1 and 10% was assessed.

### 2.7. Variant Calling and Prioritization

Data obtained after sequencing runs were analyzed using the IonTorrent Suite v.5.12.3 workflow that allows the assessment of several parameters such as Key Signal, Test Fragment, Read Length, ISP Density, and other features related to sequencing run performance. Sequences were aligned to the hg19 reference genome, and variant calling was performed using Ion Reporter version 5.18 (Thermo Fisher Scientific, Waltham, MA, USA). Variants were filtered using the following filter chain: (a) filtered coverage ≥ 100; (b) 50 ≤ allele read-count ≤ 100,000; (c) location in exonic, intronic, upstream, splicesite_5, splicesite_3; (d) minimum variant allele frequency was set to 0.05 to call somatic mutations variants; (e) variant effect in refAllele, unknown, missense, nonframeshiftInsertion, nonframeshiftDeletion, nonframeshiftBlockSubstitution, nonsense, stoploss, frameshiftInsertion, frameshiftDeletion, frameshiftBlockSubstitution; (f) 0.0 ≤ minor allele frequency ≤ 0.5; (g) SIFT score less than 0.05 or PolyPhen-2 score greater than 0.85 were used as reference scores to understand the importance of deleterious variants. Filtered variants were manually reviewed in ExAc databases, ClinVar, and gnomAD tool as well as in the scientific literature to exclude polymorphisms or nonpathogenic variants. To detect 1p/19q LOH, we firstly applied a quality criterion based on the SNP coverage. The test was considered optimal, suboptimal, or non-informative according to the number of SNPs that were covered by fewer than 250 reads. Secondly, the allelic frequencies (AF) for each SNP (with more than 250×) were annotated. SNPs were defined homozygous when the AF was approximately 100% and led the same nucleotide as that of the reference genome or having an AF of approximately 0% with a nucleotide that differed from the reference genome. SNPs with AF of approximately 50% were considered heterozygous [30]. However, taking into consideration the semi-quantitative measurement provided by NGS, we established the following confidence intervals: 0–10% or 90–100% for homozygous markers, and 40–60% for heterozygous markers. Imbalances of 1p and 19q markers due to LOH were scored when their AFs were outside the established ranges for homozygosity or heterozygosity (i.e., 10–40% or 60–90%) [30]. To identify the 1p/19q codeletion, we used the criteria described elsewhere [35,36], which established LOH as the imbalance of all informative SNPs on both chromosomal arms. More specifically, variants with heterozygous frequencies in these chromosomal arms were considered not aberrant, and a 1p/19q codeletion was defined only when no heterozygous markers were present in each arm [30]. No codeletion was scored if at least one heterozygous marker was present in 1p or 19q. A whole chromosome arm LOH was observed when no heterozygous markers were present in either arm.

## 3. Results

In this work, we developed and validated a targeted NGS approach to analyze variants or chromosomal aberrations correlated with tumorigenesis and response to treatment in gliomas.

### 3.1. Study Population

The clinical and molecular parameters of patients enrolled in the study are collected in Table 2. The median age of patients was 64 years (IQR: 54–72), with the same percentage of females and males. The median *MGMT* promoter methylation was 23% (IQR: 7–42), six patients carried out *IDH1* mutations, and seven patients showed a LOH. Notably, a value of *MGMT* promoter methylation > 9% is considered a favorable prognostic predictor, associated with a better response to therapy and therefore clinical outcome [37].

### 3.2. Target Gene Coverage

**Figure 2:**
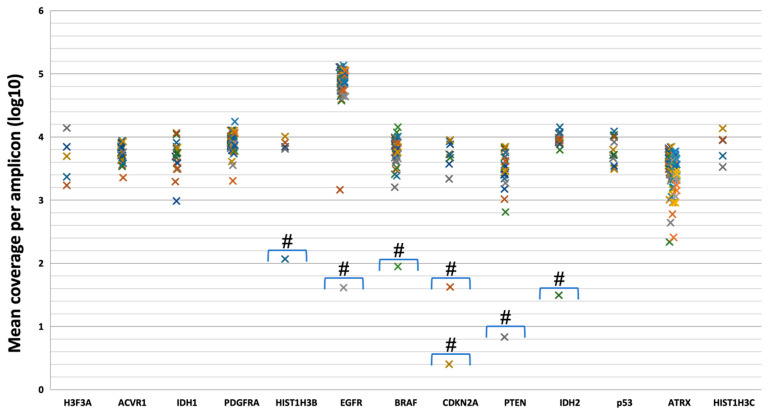
Mean coverage of amplicons of target genes. The graph shows the mean coverage of each amplicon (indicated by “×”) of 13 targeted genes in all analyzed samples. The # shows amplicons that do not satisfy a 100X depth cut-off (log10 = 2): AMPL7170424214 for *HIST1H3B*; AMPL7170319287 for *EGFR*, AMPL7170361379 for *BRAF*; AMPL7170348270 for *PTEN*; AMPL7170423161 for *IDH2*; AMPL7170325832 and AMPL7170330366 for *CDKN2A* (please refers to Supplementary Material S1, Table S2).

Targeted NGS analysis covered 13 genes (*ACVR1*, *ATRX*, *BRAF*, *CDKN2A*, *EGFR*, *H3F3A*, *HIST1H3B*, *HIST1H3C*, *IDH1*, *IDH2*, *P53*, *PDGFRA*, *PTEN*), a 125 bp region of the *TERT* promoter, and 54 SNPs located along chromosomes 1 and 19. Sequencing minimum depth was set to 100X, and, in this configuration, 97% of targeted regions achieved the minimum required cut-off. Amplicons with at least 100 reads were 98.7% and with 500 reads were 93.9%; total average coverage was 98.71% with 130 bp as mean read length and 99% of aligned reads to the hg19 reference genome. Adequate amplification efficiency was observed for 435/442 amplicons (98.4%) (mean assigned reads per amplicon Log10 ranging from three to five), while only seven amplicons (one for *HIST1H3B*, one for *EGFR*, one for *BRAF*, one for *PTEN*, one for *IDH2*, two for *CDKN2A*) had a low coverage value (Figure 2).

### 3.3. Variant Interpretation

**Figure 3:**
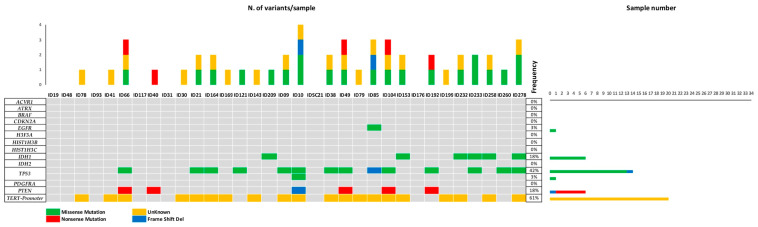
Oncoplot showing the distribution of pathogenic variants detected by tNGS in tumor samples. Each column represents a sample and each row the genes. The upper barplot shows the number of mutations in each sample, while the right barplot shows the frequency and type of mutations in each gene. The central plot shows the gene mutations in each sample. Mutation types are indicated in the legend below. The detailed list of variants is reported in Supplementary Material S1, Table S3.

The variant caller listed 2644 variants, including synonymous, unknown, missense, and indels. All variants were filtered to remove intronic variants and non-coding variants. Somatic synonymous mutations and those in the 5′ and 3′ untranslated regions (UTRs) were considered as functionally associated with cancer risk [38,39]. Using these filters, we identified 420 exonic, 1 splicesite, and 34 UTR variants, for a total of 455 alterations consisting of missense, synonymous, non-sense, nonframeshiftDeletion, and frameshiftInsertion. Based on variant allele frequency, disease prevalence, and in silico prediction score, variants were further filtered for “Pathogenic” and “Likely benign/Conflicting interpretations”. These clinically relevant variants (48) are shown in the oncoplot (Figure 3) and in Supplementary Material S1, Table S3. The frequencies of genetic variants, together with those in the TCGA database, are shown in Supplementary Material S1, Table S4.

**Figure 4:**
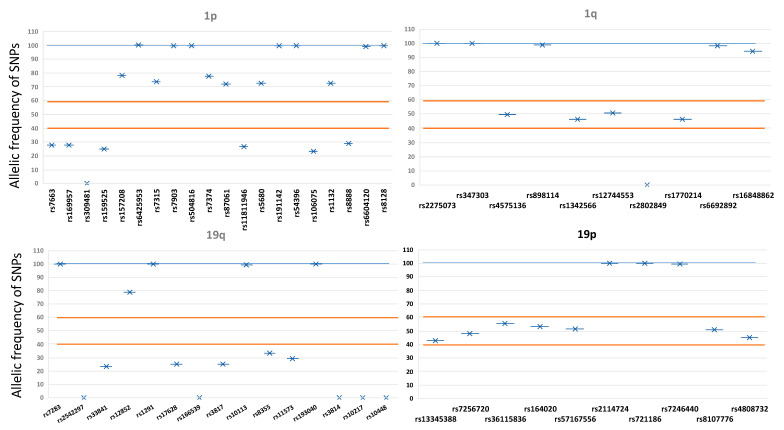
Analysis of the 1p/19q codeletion detected by the assessment of SNP allelic frequencies. The presence of all markers in 1p and 19q in homozygous form and the presence of at least one marker in heterozygous form in the opposite arm indicate codeletion of these chromosomal regions. The blue line indicates the homozygous frequency; orange lines show the established ranges of heterozygosity.

The presence of 1p/19q codeletion was assessed by a comprehensive analysis of 54 SNPs distributed across these chromosomes. As shown in Supplementary Material S2 (Figures S1 and S2), the amplicons fully covered the target regions of interest, with a good coverage value and a sufficient number of correctly mapped reads. Codeletion testing was based on the allelic frequency of each SNP, and the presence of multiple markers in 1p and 19q in the heterozygous form ruled out the presence of codeletion in these chromosomal arms. In total, we detected six samples with 1p/19q codeletion. Representative 1p/19q results are shown in Figure 4. Raw data of the tNGS are available at NCBI’s Sequence Read Archive (SRA) with the accession number SUB11581843.

### 3.4. Assessment of Sensitivity and Specificity

In order to check the performance of both the panel and the analytic bioinformatic pipeline, we measured the diagnostic accuracy, sensitivity, and specificity of the method starting from the previously known genetic information obtained by MassARRAY (Table 2). Sequencing of IDH1 revealed 6 correctly called TP samples, 28 TN samples, 0 FN, and 0 FP calls by comparing our results with expected data; thus, IDH1 sequencing resulted in an accuracy, sensitivity, and specificity of 100%. With regard to 1p/19q co-deletion, there were 6 correctly called TP samples, 27 TN samples, 1 FN (ID233), and 0 FP calls by comparing our results with expected data from MassArray, thus showing an accuracy of 97%, a sensitivity of 85%, and a specificity of 100% (please note that samples carrying only 1p or 19q deletion were considered as TN). With regard to variants located on the TERT promoter, both the two samples previously assessed by MassARRAY as carriers of TERT mutations (−146 C > T in the ID169 and the −124 C > T in the ID143, respectively) were confirmed by our workflow analysis. Moreover, the NGS panel detected 11 additional positive TERT promoter mutations in samples not previously analyzed with other technologies. For all called variants, the high proportion of concordance highlighted the high specificity of our method.

To evaluate the sensitivity of sequencing depth and derivate the LOD, DNA tumor samples with different genotype patterns were diluted with wild-type samples to create allelic frequencies between 1 and 10%. The sensitivity of variant detection was evaluated as the lowest frequency calling the somatic variant. As shown in Supplementary Material S2, for IDH1 (p.Arg132His variant), we observed a linear decrease of the allelic frequency with a lowest VAF of 2.99%. In similar experiments, we observed a VAF of 3.32% for TERT promoter (c.−146 C > T), 3.25% for PTEN (p.Arg335Ter variant), and 3.8% for TP53 (pArg282Trp variant). The variants were not called at a VAF < 2%. With regard to CNA detection limit, our method was able to identify the variant up to a 25% dilution (an example of Chr 19 deletion analysis is shown in Supplementary Material S2). Notably, in all conditions tested, the sequencing depth did not show significant variations (mean depth 1980X). Altogether, our results support the ability of our method to analyze samples with low tumor purity.

## 4. Discussion

The main objective of this study was to develop and validate a custom targeted NGS approach to identify genetic variations in key genes related to glioma tumorigenesis and treatment response. The preliminary results in terms of sequencing performances (Supplementary Material S1, Table S5), time, and costs provide encouraging evidence for the use of this approach in the clinical pipeline. This small panel size allowed for multiplexing of a large number of samples with adequate read depth. DNA input requirements were low (less than 1 ng of low-quality DNA), and the run time was considerably short (5–7 h for preparation of a pool of eight libraries with an automatic workflow and 8 h for data processing). We obtained high sequencing coverage, measured LOD, and accuracy of the method, and we detected a substantial number of clinically relevant variants in each analyzed sample. The amplicon-based method provided a detailed genetic characterization of genes currently required for molecular testing in gliomas. The method used in this study confirmed and extended previous molecular profiling performed by the MassARRAY Analyzer 4 system (Agena Bioscience, San Diego, CA, USA) (Table 2 and Section 2).

Below, we introduce each of the genes included in the custom panel for their relevance in the diagnosis and management of patients with gliomas and briefly describe the identified genetic variants that are associated with adverse outcomes, disease progression, or drug resistance according to ClinVar (Supplementary Material S1, Table S3).

Isocitrate dehydrogenase genes (*IDH1* and *IDH2)* are frequently associated with anaplastic astrocytic, oligodendroglial, and lower grade tumors [40]. IDH enzymes are involved in several cellular processes, including mitochondrial oxidative phosphorylation, glutamate metabolism, lipogenesis, regulation of glucose, DNA repair, and cellular energy balance [41]. The main heterozygous somatic point mutations are localized in the active sites of the both IDH enzymes. Using our panel, we confirmed the presence of pathogenic missense variant p.R132H in *IDH1* in six patients. This point mutation in exon 4 involves arginine in position 132 and its homolog at codon 172 of *IDH2* [42,43]. Deregulated IDH activity results in the inhibition of key enzymes involved in demethylation of histones and DNA, with hypermethylation, altered gene expression, and proliferation of mutant cells [44,45]. The prognostic significance of the *IDH1* R132H mutation is inconsistent and was associated with tumor WHO grade. Several studies demonstrated an association between an IDH1 mutation and improved prognosis OS, whereas others showed no significant difference [46]. Several pieces of evidence have shown that *IDH1/IDH2* mutational status is an important diagnostic and prognostic factor to predict the response to anti-IDH treatment [47]. In clinical trials, glioblastoma patients with *IDH1* R132H treated with vandetanib or ivosidenib in combination with temozolomide showed a significant increase in overall survival (OS) compared to glioblastoma patients without *IDH1* R132H [48,49,50].

*EGFR* belongs to the ErbB receptor family and is one of the major factors in glioma tumorigenesis [51]. EGFR plays a role in cell growth and proliferation, autophagy, and cell metabolism. Located on chromosomal band 7p12, known *EGFR* genetic alterations include point mutations, rearrangement, and copy number variations [52]. In gliomas, *EGFR* amplification promotes invasion, proliferation, and failure of radiotherapy and chemotherapy [53]. In our cohort, we identified the missense variant p.Gly719Ala in exon 18 with pathogenic/likely pathogenic clinical significance according to ClinVar (Supplementary Material S1, Table S3). In My Cancer Genomics, the presence of an EGFR-G719A mutation is cited as an inclusion criterion for glioblastoma clinical trials. In particular, a phase II trial involving patients with EGFR-activated glioblastoma is evaluating the effect of osimertinib on tumor cell growth by blocking enzymes required for cell growth [54,55].

Genetic alterations in the *TERT* promoter are the most frequent non-coding mutations and the earliest genetic events that occur in GBM development [56]. Germline and somatic alterations in the promoter region of this gene are point mutations, genomic rearrangements, and DNA amplifications that affect telomerase activity [57]. *TERT*p mutations, although not reported in ClinVar, are associated with a better prognosis in *IDH*-mutated gliomas, and a worse prognosis in patients with primary GBM [58]. In our cohort, two hotspot mutations, c.−124C > T and c.−146C > T, were identified (Supplementary Material S1, Table S3). These somatic mutations are known to increase *TERT* expression, telomerase enzyme activity, telomere length, and tumor formation. Although TERT mutation prevalence is over 80% in gliomas, their role as a prognostic/predictive biomarker is still largely controversial [59]. Numerous studies indicated the *TERT* promoter mutation as an independent factor for poor prognosis, and various approaches to target TERT activity, such as inhibitors, immunotherapy, and vaccines, are currently being explored [60]. Several clinical trials are evaluating the efficacy of surgery and temozolomide chemotherapy in combination with TERT inhibitors such as eribulin, imetelstat, and BIBR1532 [56,60].

*TP53* is one of the most deregulated genes in neoplasms and is involved in cell cycle and invasion; response to DNA damage and immune response; cell differentiation; and proliferation, apoptosis, and genomic stability [61]. The high expression of gain of function (GOF) oncogenic variants of the p53 protein is associated with survival outcomes and with a more invasive and aggressive phenotype [61]. In our cohort, we identified several missense variants in *TP53* with pathogenic/likely pathogenic clinical significance (Supplementary Material S1, Table S3) and some previously associated with gliomas [62,63,64].

*ATRX* encodes a chromatin-remodeling protein whose main function correlates with gene expression regulation [65]. *ATRX* mutations are strongly linked to DNA damage and apoptosis. Its alterations includes point mutations or deletions and are correlated with the Alternative Lengthening of Telomeres (ALT) phenotype, *TP53* mutations, *MGMT* methylation status, and 1p/19q co-deletion [66]. In particular, loss of *ATRX* leads to increased genome instability and micronuclei formation, which can influence the response to therapy and the prognosis [67,68].

*PTEN* is a tumor suppressor gene that plays an important role in regulating cell cycle progression, cell migration, apoptosis, and DNA damage repair [69]. In gliomas, loss of *PTEN* expression has been associated with increased malignancy, metastasis, radiotherapy, and chemotherapy response [70]. In our cohort of patients, we identified four *PTEN* variants (c.697 C > T, c.1003 C > T, c.822G > A, and c.972delT), which are predicted to cause loss of normal protein function and therefore are classified as pathogenic and disease-causing (Supplementary Material S1, Table S3).

*PDGFRA* encodes for a cell-surface transmembrane receptor that controls many important cellular processes such as normal proliferation, growth, proliferation, and survival of glial cells [71,72]. Several genetic rearrangements in *PDGFRA* have been identified in gliomas, but their diagnostic or prognostic significance is unclear [73].

Activin A receptor type I (*ACVR1*) encodes a bone morphogenetic receptor. Its function has been linked to many biological processes and, recently, to tumorigenesis [74]. Somatic mutations in *ACVR1* have been associated with high-grade pediatric brainstem gliomas, but their specific role remains to be elucidated [75].

*BRAF* plays key roles in proliferation, differentiation, cell motility, and apoptosis [76]. Based on these findings, the use of *BRAF* as a therapeutic target has been a milestone [77]. One of targetable mutations is *BRAF*, i.e., p.V600E, which occurs in roughly half of all epithelioid gliomas but is rare in classic GBM [78]. Detection of the *BRAF* V600E mutation represents a positive prognostic indicator [78].

Mutations in *H3F3A* and in *HIST1H3B/C* genes play a pivotal role in development of diffuse midline glioma, as well as a subset of adult glioblastoma multiforme [79]. The recurrent K27M mutations in both genes arising in the thalamus, brainstem, and spinal cord are associated with poor prognosis regardless of histologic grade [80]. In contrast, p.G34R or p.G34V mutations in *H3F3A* are found in the cerebral cortex of adolescent and young adults and are associated with a better prognosis [81].

Complete deletion of both the short arm of chromosome 1 (1p) and the long arm of chromosome 19 (19q) (1p/19q-codeletion) is a genetic signature occurring in the pathogenesis of oligodendrogliomas [82]. This 1p/19q co-deletion is a favorable predictive biomarker for response to alkylating chemotherapy, either alone or in combination with radiotherapy. Several pieces of evidence show a correlation between the LOH in 1p/19q and the mutational status of *IDH1/2*, *TERT*, and *MGMT*. Here we used an approach to identify the codeletion in these chromosomal arms based on the analysis of 54 SNPs, which enabled the identification of LOH in six patients.

The present study, using a targeted sequencing method, enabled a comprehensive genetic profile of each sample tested. The diagnostic and therapeutic benefits for the use of this approach are numerous. The possibility to consider more effective therapies associated with molecular alterations represents one of the most important innovations in oncology. Several tumors are characterized by a high degree of heterogeneity, and their primitive molecular profile may also change in response to ongoing treatments. Compared to other forms of brain tumors, gliomas are the most heterogeneous in terms of molecular profile and prognosis [83]. Therefore, the diagnostic approach should include high-throughput methods, such as NGS, to investigate the presence of multiple molecular alterations [84]. In addition to the benefits for patients, the advantages of NGS also rely on the reduction of time to obtain results and reduced costs. The economic advantage of the NGS-based approach versus traditional diagnostic approaches (Sanger sequencing or Real-Time PCR) has been already discussed [85]. Targeted sequencing, in particular, may allow a significant reduction in costs and simplification of clinical interpretation of the identified genotypes. Systematic profiling of the comprehensive set of mutations related to glioma may offer new clinical opportunities for tailored treatments, shorter turnaround times, and less labor intensive and more affordable testing.

## 5. Conclusions

The goal of the genomic approach proposed here is to better characterize the molecular portrait of gliomas, enabling faster tumor classification and better genotype–phenotype characterization. This high-throughput approach may enable personalized medicine and contribute to a better healthcare with a cost reduction.

## Figures and Tables

**Table 1:** Target regions of the NGS panel.

Target	Chromosome	Number of Amplicons	Covered Bases	% Overall Coverage	Number of Exons
*ACVR1*	chr2	23	1710	100	9
*ATRX*	chrX	106	8161	99	35
*BRAF*	chr7	42	2655	99	18
*CDKN2A*	chr9	11	1008	99	5
*EGFR*	chr7	63	4489	100	30
*H3F3A*	chr1	5	273	58	3
*HIST1H3B*	chr6	5	431	100	1
*HIST1H3C*	chr6	5	431	100	1
*IDH1*	chr2	20	1405	100	8
*IDH2*	chr15	20	1572	99	11
*TP53*	chr17	13	1503	100	12
*PDGFRA*	chr4	50	3710	100	22
*PTEN*	chr10	22	1901	98	10
*TERT-Promoter*	chr5	1	124	99	n/a
29 SNPs	chr1	30	30	100	n/a
25 SNPs	chr19	24	24	100	n/a

**Table 2:** Clinical and molecular characteristics of patients and related tumor WHO grade and location.

Sample ID	WHO Grade	Tumor Location	Sex	Age	MGMT	*Idh1/2* ^1^	1p/19q LOH ^1^	*TERT* ^1^	Ki-67 (MIB-1)
ID 19	IV	Right TI	M	67	4%	wt	wt	-	25%
ID 48	IV	Right F	M	72	8%	wt	wt	-	20%
ID 78	IV	Right T	F	67	22%	wt	wt	-	17%
ID 93	IV	Right F	M	62	44%	wt	wt	-	35%
ID 41	IV	Right TI	F	45	23%	wt	wt	-	37%
ID 66	IV	Right T	M	47	63%	wt	wt	-	30%
ID 117	IV	Right FTP	F	56	67%	wt	wt	-	30%
ID 169	IV	Right F	M	52	13%	wt	wt	–146 C > T	67%
ID 121	IV	Right P	M	50	26%	wt	wt	-	60%
ID 143	IV	Right F	M	61	23%	wt	wt	–124 C > T	30%
ID 31	IV	Left FP	M	75	4%	wt	wt	-	25%
ID 30	IV	Left PO	F	76	41%	wt	wt	-	-
ID 21	IV	Right P	F	54	49%	wt	wt	-	80%
ID 164	IV	Right F	F	63	2%	wt	wt	-	40%
ID 40	IV	Right T	F	76	27%	wt	wt	-	35%
ID 166	IV	Left T	F	73	4%	wt	wt	-	-
ID 153	III	Right F	F	67	72%	p.R132H	LOH	-	4%
ID 209	IV	Left P	M	74	56%	p.R132H	LOH	-	70%
ID 232	II	Left F	F	48	53%	p.R132H	LOH	-	5%
ID 233	IV	Right T	M	42	60%	p.R132H	LOH	-	20%
ID 258	II	Left T	M	47	33%	p.R132H	LOH	-	4%
ID 260	IV	Left CC	M	41	5%	wt	LOH	-	-
ID 278	II	Left F	F	52	35%	p.R132H	LOH	-	1%
ID 49	IV	Left F	M	51	32%	wt	wt	-	50%
ID 79	IV	Right P	F	71	28%	wt	wt	-	20%
ID 38	IV	Right PO	M	54	5%	wt	wt	-	20%
ID 192	IV	Left T	F	91	-	wt	wt	-	-
ID 199	IV	Right F	M	53	-	wt	wt	-	-
ID 176	IV	Right F	M	62	3%	wt	wt	-	-
ID 104	IV	Right F	F	70	3%	wt	wt	-	85%
ID 09	IV	Left FP	F	81	4%	wt	1p	-	15%
ID 10	IV	Right FI	F	63	9%	wt	19q	-	35%
ID 85	IV	Left P	M	59	4%	wt	19q	-	30%
ID sc21	IV	Right P	M	46	41%	wt	1p	-	60%

T = temporal lobe; I = insular lobe; F = frontal lobe; P = parietal lobe; O = occipital lobe; - = test not performed; M = Male; F = Female. ^1^ The molecular profile was performed by the MassARRAY Analyzer 4 system (Agena Bioscience, CA, USA).

## Data Availability

Not applicable.

## Supplementary Materials

The following supporting information can be downloaded at: www.mdpi.com/article/10.3390/life12070956/s1, Figure S1: Amplicons’ coverage of chr1. The graph shows the 29 amplicons used to assess copy number alterations of chr1; Figure S2: Amplicons’ coverage of chr19. The graph shows the 25 amplicons used to assess copy number alterations of chr19; Table S1: On-demand NGS panel designed using the custom IonAmpliSeq Designer software; Table S2: Designed BED file; Table S3: Summary of clinically relevant variants identified in our cohort; Table S4: Frequencies of genetic variants according TCGA database; Table S5: NGS summary report.

## Abbreviations

AF	Allelic frequencies
ALT	Alternative lengthening of telomeres
AML	Acute myeloid leukemia
AMP–ASCO–CAP	Association for Molecular Pathology, AMP; American Society of Clinical Oncology, ASCO; and College of American Pathologists, CAP
ATRX	α thalassemia/mental retardation syndrome X-linked gene
BRAF	B-Raf proto-oncogene
cIMPACT	Consortium to Inform Molecular and Practical Approaches to CNS Tumor Taxonomy
CNAs	Copy number alterations
COSMIC	Catalogue OF Somatic Mutations In Cancer
EGFR	Epidermal growth factor receptor
GBM	Glioblastoma
GDC	Genomic Data Commons
GOF	Gain of function
IARC	International Agency for Research on Cancer
IDH	Isocitrate dehydrogenase
LOH	Loss of heterozygosity
MGMT	O6-methylguanine (O6-MeG)-DNA methyltransferase
NGS	Next generation sequencing
OS	Overall survival
PDGFR	Platelet-derived growth factor receptor
PTEN	Phosphatase and tensin Homolog
SNVs	Single nucleotide variants
TCGA	Cancer Genome Atlas
TERT	Telomerase reverse transcriptase
TMZ	Temozolomide
TP53	Tumor protein P53
WHO	World Health Organization

## References

[1] Yoon N., Kim H.S., Lee J.W., Lee E.J., Maeng L.S., Yoon W.S. (2020). Targeted Genomic Sequencing Reveals Different Evolutionary Patterns Between Locally and Distally Recurrent Glioblastomas. Cancer Genom. Proteom..

[2] Sakthikumar S., Roy A., Haseeb L., Pettersson M.E., Sundstrom E., Marinescu V.D., Lindblad-Toh K., Forsberg-Nilsson K. (2020). Whole-genome sequencing of glioblastoma reveals enrichment of non-coding constraint mutations in known and novel genes. Genome Biol..

[3] Pesenti C., Navone S.E., Guarnaccia L., Terrasi A., Costanza J., Silipigni R., Guarneri S., Fusco N., Fontana L., Locatelli M. (2019). The Genetic Landscape of Human Glioblastoma and Matched Primary Cancer Stem Cells Reveals Intratumour Similarity and Intertumour Heterogeneity. Stem Cells Int..

[4] Ohgaki H., Kleihues P. (2013). The definition of primary and secondary glioblastoma. Clin. Cancer Res..

[5] Campanella R., Guarnaccia L., Caroli M., Zarino B., Carrabba G., La Verde N., Gaudino C., Rampini A., Luzzi S., Riboni L. (2020). Personalized and translational approach for malignant brain tumors in the era of precision medicine: The strategic contribution of an experienced neurosurgery laboratory in a modern neurosurgery and neuro-oncology department. J. Neurol. Sci..

[6] Verhaak R.G., Hoadley K.A., Purdom E., Wang V., Qi Y., Wilkerson M.D., Miller C.R., Ding L., Golub T., Mesirov J.P. (2010). Integrated genomic analysis identifies clinically relevant subtypes of glioblastoma characterized by abnormalities in PDGFRA, IDH1, EGFR, and NF1. Cancer Cell.

[7] Behnan J., Finocchiaro G., Hanna G. (2019). The landscape of the mesenchymal signature in brain tumours. Brain.

[8] Cohen A.L., Holmen S.L., Colman H. (2013). IDH1 and IDH2 mutations in gliomas. Curr. Neurol. Neurosci. Rep..

[9] Woo H.Y., Na K., Yoo J., Chang J.H., Park Y.N., Shim H.S., Kim S.H. (2020). Glioblastomas harboring gene fusions detected by next-generation sequencing. Brain Tumor Pathol..

[10] Ceccarelli M., D’Andrea G., Micheli L., Gentile G., Cavallaro S., Merlino G., Papoff G., Tirone F. (2021). Tumor Growth in the High Frequency Medulloblastoma Mouse Model Ptch1(+/−)/Tis21(KO) Has a Specific Activation Signature of the PI3K/AKT/mTOR Pathway and Is Counteracted by the PI3K Inhibitor MEN1611. Front. Oncol..

[11] Blomquist M.R., Ensign S.F., D’Angelo F., Phillips J.J., Ceccarelli M., Peng S., Halperin R.F., Caruso F.P., Garofano L., Byron S.A. (2020). Temporospatial genomic profiling in glioblastoma identifies commonly altered core pathways underlying tumor progression. Neurooncol. Adv..

[12] Louis D.N., Perry A., Wesseling P., Brat D.J., Cree I.A., Figarella-Branger D., Hawkins C., Ng H.K., Pfister S.M., Reifenberger G. (2021). The 2021 WHO Classification of Tumors of the Central Nervous System: A summary. Neuro Oncol..

[13] Brennan C.W., Verhaak R.G., McKenna A., Campos B., Noushmehr H., Salama S.R., Zheng S., Chakravarty D., Sanborn J.Z., Berman S.H. (2013). The somatic genomic landscape of glioblastoma. Cell.

[14] Gadji M., Fortin D., Tsanaclis A.M., Drouin R. (2009). Is the 1p/19q deletion a diagnostic marker of oligodendrogliomas?. Cancer Genet. Cytogenet..

[15] Lin Y., Xing Z., She D., Yang X., Zheng Y., Xiao Z., Wang X., Cao D. (2017). IDH mutant and 1p/19q co-deleted oligodendrogliomas: Tumor grade stratification using diffusion-, susceptibility-, and perfusion-weighted MRI. Neuroradiology.

[16] Stichel D., Ebrahimi A., Reuss D., Schrimpf D., Ono T., Shirahata M., Reifenberger G., Weller M., Hanggi D., Wick W. (2018). Distribution of EGFR amplification, combined chromosome 7 gain and chromosome 10 loss, and TERT promoter mutation in brain tumors and their potential for the reclassification of IDHwt astrocytoma to glioblastoma. Acta Neuropathol..

[17] Crespo I., Vital A.L., Nieto A.B., Rebelo O., Tao H., Lopes M.C., Oliveira C.R., French P.J., Orfao A., Tabernero M.D. (2011). Detailed characterization of alterations of chromosomes 7, 9, and 10 in glioblastomas as assessed by single-nucleotide polymorphism arrays. J. Mol. Diagn..

[18] Tan J.Y., Wijesinghe I.V.S., Alfarizal Kamarudin M.N., Parhar I. (2021). Paediatric Gliomas: BRAF and Histone H3 as Biomarkers, Therapy and Perspective of Liquid Biopsies. Cancers.

[19] Villa C., Miquel C., Mosses D., Bernier M., Di Stefano A.L. (2018). The 2016 World Health Organization classification of tumours of the central nervous system. Presse Med..

[20] Komori T. (2021). Grading of adult diffuse gliomas according to the 2021 WHO Classification of Tumors of the Central Nervous System. Lab. Investig..

[21] Zhang P., Xia Q., Liu L., Li S., Dong L. (2020). Current Opinion on Molecular Characterization for GBM Classification in Guiding Clinical Diagnosis, Prognosis, and Therapy. Front. Mol. Biosci..

[22] Theeler B.J., Gilbert M.R. (2015). Advances in the treatment of newly diagnosed glioblastoma. BMC Med..

[23] Yang J., Wang L., Xu Z., Wu L., Liu B., Wang J., Tian D., Xiong X., Chen Q. (2020). Integrated Analysis to Evaluate the Prognostic Value of Signature mRNAs in Glioblastoma Multiforme. Front. Genet..

[24] Weller M., van den Bent M., Preusser M., Le Rhun E., Tonn J.C., Minniti G., Bendszus M., Balana C., Chinot O., Dirven L. (2021). EANO guidelines on the diagnosis and treatment of diffuse gliomas of adulthood. Nat. Rev. Clin. Oncol..

[25] Guan Y.F., Li G.R., Wang R.J., Yi Y.T., Yang L., Jiang D., Zhang X.P., Peng Y. (2012). Application of next-generation sequencing in clinical oncology to advance personalized treatment of cancer. Chin. J. Cancer.

[26] Lorenz J., Rothhammer-Hampl T., Zoubaa S., Bumes E., Pukrop T., Kolbl O., Corbacioglu S., Schmidt N.O., Proescholdt M., Hau P. (2020). A comprehensive DNA panel next generation sequencing approach supporting diagnostics and therapy prediction in neurooncology. Acta Neuropathol. Commun..

[27] Sahm F., Schrimpf D., Jones D.T., Meyer J., Kratz A., Reuss D., Capper D., Koelsche C., Korshunov A., Wiestler B. (2016). Next-generation sequencing in routine brain tumor diagnostics enables an integrated diagnosis and identifies actionable targets. Acta Neuropathol..

[28] Wesseling P., Capper D. (2018). WHO 2016 Classification of gliomas. Neuropathol. Appl. Neurobiol..

[29] Stupp R., Taillibert S., Kanner A.A., Kesari S., Steinberg D.M., Toms S.A., Taylor L.P., Lieberman F., Silvani A., Fink K.L. (2015). Maintenance Therapy With Tumor-Treating Fields Plus Temozolomide vs Temozolomide Alone for Glioblastoma: A Randomized Clinical Trial. JAMA.

[30] D’Haene N., Melendez B., Blanchard O., De Neve N., Lebrun L., Van Campenhout C., Salmon I. (2019). Design and Validation of a Gene-Targeted, Next-Generation Sequencing Panel for Routine Diagnosis in Gliomas. Cancers.

[31] Trevethan R. (2017). Sensitivity, Specificity, and Predictive Values: Foundations, Pliabilities, and Pitfalls in Research and Practice. Front. Public Health.

[32] Jennings L.J., Arcila M.E., Corless C., Kamel-Reid S., Lubin I.M., Pfeifer J., Temple-Smolkin R.L., Voelkerding K.V., Nikiforova M.N. (2017). Guidelines for Validation of Next-Generation Sequencing-Based Oncology Panels: A Joint Consensus Recommendation of the Association for Molecular Pathology and College of American Pathologists. J. Mol. Diagn..

[33] Singh R.R. (2020). Next-Generation Sequencing in High-Sensitive Detection of Mutations in Tumors: Challenges, Advances, and Applications. J. Mol. Diagn..

[34] Dagogo-Jack I., Shaw A.T. (2018). Tumour heterogeneity and resistance to cancer therapies. Nat. Rev. Clin. Oncol..

[35] Dubbink H.J., Atmodimedjo P.N., van Marion R., Krol N.M.G., Riegman P.H.J., Kros J.M., van den Bent M.J., Dinjens W.N.M. (2016). Diagnostic Detection of Allelic Losses and Imbalances by Next-Generation Sequencing: 1p/19q Co-Deletion Analysis of Gliomas. J. Mol. Diagn..

[36] Hatanpaa K.J., Burger P.C., Eshleman J.R., Murphy K.M., Berg K.D. (2003). Molecular diagnosis of oligodendroglioma in paraffin sections. Lab. Investig..

[37] Fontana L., Tabano S., Bonaparte E., Marfia G., Pesenti C., Falcone R., Augello C., Carlessi N., Silipigni R., Guerneri S. (2016). MGMT-Methylated Alleles Are Distributed Heterogeneously Within Glioma Samples Irrespective of IDH Status and Chromosome 10q Deletion. J. Neuropathol. Exp. Neurol..

[38] Zhang D., Xia J. (2020). Somatic synonymous mutations in regulatory elements contribute to the genetic aetiology of melanoma. BMC Med. Genom..

[39] Gotea V., Gartner J.J., Qutob N., Elnitski L., Samuels Y. (2015). The functional relevance of somatic synonymous mutations in melanoma and other cancers. Pigment Cell Melanoma Res..

[40] Qi S., Yu L., Li H., Ou Y., Qiu X., Ding Y., Han H., Zhang X. (2014). Isocitrate dehydrogenase mutation is associated with tumor location and magnetic resonance imaging characteristics in astrocytic neoplasms. Oncol. Lett..

[41] Reitman Z.J., Yan H. (2010). Isocitrate dehydrogenase 1 and 2 mutations in cancer: Alterations at a crossroads of cellular metabolism. J. Natl. Cancer Inst..

[42] Starkova J., Hermanova I., Hlozkova K., Hararova A., Trka J. (2018). Altered Metabolism of Leukemic Cells: New Therapeutic Opportunity. Int. Rev. Cell Mol. Biol..

[43] Berger M.S., Hervey-Jumper S., Wick W. (2016). Astrocytic gliomas WHO grades II and III. Handb. Clin. Neurol..

[44] Tommasini-Ghelfi S., Murnan K., Kouri F.M., Mahajan A.S., May J.L., Stegh A.H. (2019). Cancer-associated mutation and beyond: The emerging biology of isocitrate dehydrogenases in human disease. Sci. Adv..

[45] Yang H., Ye D., Guan K.L., Xiong Y. (2012). IDH1 and IDH2 mutations in tumorigenesis: Mechanistic insights and clinical perspectives. Clin. Cancer Res..

[46] Mu L., Xu W., Li Q., Ge H., Bao H., Xia S., Ji J., Jiang J., Song Y., Gao Q. (2017). IDH1 R132H Mutation Is Accompanied with Malignant Progression of Paired Primary-Recurrent Astrocytic Tumours. J. Cancer.

[47] Guo J., Zhang R., Yang Z., Duan Z., Yin D., Zhou Y. (2021). Biological Roles and Therapeutic Applications of IDH2 Mutations in Human Cancer. Front. Oncol..

[48] Lee E.Q., Kaley T.J., Duda D.G., Schiff D., Lassman A.B., Wong E.T., Mikkelsen T., Purow B.W., Muzikansky A., Ancukiewicz M. (2015). A Multicenter, Phase II, Randomized, Noncomparative Clinical Trial of Radiation and Temozolomide with or without Vandetanib in Newly Diagnosed Glioblastoma Patients. Clin. Cancer Res..

[49] DiNardo C.D., Stein A.S., Stein E.M., Fathi A.T., Frankfurt O., Schuh A.C., Dohner H., Martinelli G., Patel P.A., Raffoux E. (2021). Mutant Isocitrate Dehydrogenase 1 Inhibitor Ivosidenib in Combination With Azacitidine for Newly Diagnosed Acute Myeloid Leukemia. J. Clin. Oncol..

[50] Mellinghoff I.K., Ellingson B.M., Touat M., Maher E., De La Fuente M.I., Holdhoff M., Cote G.M., Burris H., Janku F., Young R.J. (2020). Ivosidenib in Isocitrate Dehydrogenase 1-Mutated Advanced Glioma. J. Clin. Oncol..

[51] Sigismund S., Avanzato D., Lanzetti L. (2018). Emerging functions of the EGFR in cancer. Mol. Oncol..

[52] Saadeh F.S., Mahfouz R., Assi H.I. (2018). EGFR as a clinical marker in glioblastomas and other gliomas. Int. J. Biol. Markers.

[53] Pan P.C., Magge R.S. (2020). Mechanisms of EGFR Resistance in Glioblastoma. Int. J. Mol. Sci..

[54] Makhlin I., Salinas R.D., Zhang D., Jacob F., Ming G.L., Song H., Saxena D., Dorsey J.F., Nasrallah M.P., Morrissette J.J. (2019). Clinical activity of the EGFR tyrosine kinase inhibitor osimertinib in EGFR-mutant glioblastoma. CNS Oncol..

[55] Chagoya G., Kwatra S.G., Nanni C.W., Roberts C.M., Phillips S.M., Nullmeyergh S., Gilmore S.P., Spasojevic I., Corcoran D.L., Young C.C. (2020). Efficacy of osimertinib against EGFRvIII+ glioblastoma. Oncotarget.

[56] Olympios N., Gilard V., Marguet F., Clatot F., Di Fiore F., Fontanilles M. (2021). TERT Promoter Alterations in Glioblastoma: A Systematic Review. Cancers.

[57] Vinagre J., Almeida A., Populo H., Batista R., Lyra J., Pinto V., Coelho R., Celestino R., Prazeres H., Lima L. (2013). Frequency of TERT promoter mutations in human cancers. Nat. Commun..

[58] Jeong D.E., Woo S.R., Nam H., Nam D.H., Lee J.H., Joo K.M. (2017). Preclinical and clinical implications of TERT promoter mutation in glioblastoma multiforme. Oncol. Lett..

[59] Rachakonda P.S., Hosen I., de Verdier P.J., Fallah M., Heidenreich B., Ryk C., Wiklund N.P., Steineck G., Schadendorf D., Hemminki K. (2013). TERT promoter mutations in bladder cancer affect patient survival and disease recurrence through modification by a common polymorphism. Proc. Natl. Acad. Sci. USA.

[60] Amen A.M., Fellmann C., Soczek K.M., Ren S.M., Lew R.J., Knott G.J., Park J.E., McKinney A.M., Mancini A., Doudna J.A. (2021). Cancer-specific loss of TERT activation sensitizes glioblastoma to DNA damage. Proc. Natl. Acad. Sci. USA.

[61] Zhang Y., Dube C., Gibert M., Cruickshanks N., Wang B., Coughlan M., Yang Y., Setiady I., Deveau C., Saoud K. (2018). The p53 Pathway in Glioblastoma. Cancers.

[62] Hanel W., Marchenko N., Xu S., Yu S.X., Weng W., Moll U. (2013). Two hot spot mutant p53 mouse models display differential gain of function in tumorigenesis. Cell Death Differ..

[63] Wong P., Verselis S.J., Garber J.E., Schneider K., DiGianni L., Stockwell D.H., Li F.P., Syngal S. (2006). Prevalence of early onset colorectal cancer in 397 patients with classic Li-Fraumeni syndrome. Gastroenterology.

[64] Manoharan V., Karunanayake E.H., Tennekoon K.H., De Silva S., Imthikab A.I.A., De Silva K., Angunawela P., Vishwakula S., Lunec J. (2020). Pattern of nucleotide variants of TP53 and their correlation with the expression of p53 and its downstream proteins in a Sri Lankan cohort of breast and colorectal cancer patients. BMC Cancer.

[65] De Luca C., Race V., Keldermans L., Bauters M., Van Esch H. (2020). Challenges in molecular diagnosis of X-linked Intellectual disability. Br. Med. Bull..

[66] Nandakumar P., Mansouri A., Das S. (2017). The Role of ATRX in Glioma Biology. Front. Oncol..

[67] Haase S., Garcia-Fabiani M.B., Carney S., Altshuler D., Nunez F.J., Mendez F.M., Nunez F., Lowenstein P.R., Castro M.G. (2018). Mutant ATRX: Uncovering a new therapeutic target for glioma. Expert Opin. Ther. Targets.

[68] Qin T., Mullan B., Ravindran R., Messinger D., Siada R., Cummings J.R., Harris M., Muruganand A., Pyaram K., Miklja Z. (2022). ATRX loss in glioma results in dysregulation of cell-cycle phase transition and ATM inhibitor radio-sensitization. Cell Rep..

[69] Benitez J.A., Ma J., D’Antonio M., Boyer A., Camargo M.F., Zanca C., Kelly S., Khodadadi-Jamayran A., Jameson N.M., Andersen M. (2017). PTEN regulates glioblastoma oncogenesis through chromatin-associated complexes of DAXX and histone H3.3. Nat. Commun..

[70] Han F., Hu R., Yang H., Liu J., Sui J., Xiang X., Wang F., Chu L., Song S. (2016). PTEN gene mutations correlate to poor prognosis in glioma patients: A meta-analysis. Onco Targets Ther..

[71] Ozawa T., Brennan C.W., Wang L., Squatrito M., Sasayama T., Nakada M., Huse J.T., Pedraza A., Utsuki S., Yasui Y. (2010). PDGFRA gene rearrangements are frequent genetic events in PDGFRA-amplified glioblastomas. Genes Dev..

[72] Alentorn A., Marie Y., Carpentier C., Boisselier B., Giry M., Labussiere M., Mokhtari K., Hoang-Xuan K., Sanson M., Delattre J.Y. (2012). Prevalence, clinico-pathological value, and co-occurrence of PDGFRA abnormalities in diffuse gliomas. Neuro Oncol..

[73] Cantanhede I.G., de Oliveira J.R.M. (2017). PDGF Family Expression in Glioblastoma Multiforme: Data Compilation from Ivy Glioblastoma Atlas Project Database. Sci. Rep..

[74] Taylor K.R., Vinci M., Bullock A.N., Jones C. (2014). ACVR1 mutations in DIPG: Lessons learned from FOP. Cancer Res..

[75] Fortin J., Tian R., Zarrabi I., Hill G., Williams E., Sanchez-Duffhues G., Thorikay M., Ramachandran P., Siddaway R., Wong J.F. (2020). Mutant ACVR1 Arrests Glial Cell Differentiation to Drive Tumorigenesis in Pediatric Gliomas. Cancer Cell.

[76] Ascierto P.A., Kirkwood J.M., Grob J.J., Simeone E., Grimaldi A.M., Maio M., Palmieri G., Testori A., Marincola F.M., Mozzillo N. (2012). The role of BRAF V600 mutation in melanoma. J. Transl. Med..

[77] Ye P., Cai P., Xie J., Zhang J. (2021). Reliability of BRAF mutation detection using plasma sample: A systematic review and meta-analysis. Medicine.

[78] Natsumeda M., Chang M., Gabdulkhaev R., Takahashi H., Tsukamoto Y., Kanemaru Y., Okada M., Oishi M., Okamoto K., Rodriguez F.J. (2021). Predicting BRAF V600E mutation in glioblastoma: Utility of radiographic features. Brain Tumor Pathol..

[79] Castel D., Philippe C., Calmon R., Le Dret L., Truffaux N., Boddaert N., Pages M., Taylor K.R., Saulnier P., Lacroix L. (2015). Histone H3F3A and HIST1H3B K27M mutations define two subgroups of diffuse intrinsic pontine gliomas with different prognosis and phenotypes. Acta Neuropathol..

[80] Sloan E.A., Cooney T., Oberheim Bush N.A., Buerki R., Taylor J., Clarke J.L., Torkildson J., Kline C., Reddy A., Mueller S. (2019). Recurrent non-canonical histone H3 mutations in spinal cord diffuse gliomas. Acta Neuropathol..

[81] Haase S., Nunez F.M., Gauss J.C., Thompson S., Brumley E., Lowenstein P., Castro M.G. (2020). Hemispherical Pediatric High-Grade Glioma: Molecular Basis and Therapeutic Opportunities. Int. J. Mol. Sci..

[82] Mizoguchi M., Yoshimoto K., Ma X., Guan Y., Hata N., Amano T., Nakamizo A., Suzuki S.O., Iwaki T., Sasaki T. (2012). Molecular characteristics of glioblastoma with 1p/19q co-deletion. Brain Tumor Pathol..

[83] Conway J.R., Warner J.L., Rubinstein W.S., Miller R.S. (2019). Next-Generation Sequencing and the Clinical Oncology Workflow: Data Challenges, Proposed Solutions, and a Call to Action. JCO Precis. Oncol..

[84] Ozretic L., Heukamp L.C., Odenthal M., Buettner R. (2012). The role of molecular diagnostics in cancer diagnosis and treatment. Onkologie.

[85] Pruneri G., De Braud F., Sapino A., Aglietta M., Vecchione A., Giusti R., Marchio C., Scarpino S., Baggi A., Bonetti G. (2021). Next-Generation Sequencing in Clinical Practice: Is It a Cost-Saving Alternative to a Single-Gene Testing Approach?. Pharm. Open.

