# Supplementary material for: A Targeted Next-Generation Sequencing Panel to Genotype Gliomas
Source: Life (Basel). 2022 Jun 24;12(7):956. doi: 10.3390/life12070956 (PMC9320073; doi:10.3390/life12070956)
Supplement: Supplementary file 1 [file life-12-00956-s001.zip › Supplementary materials S2.pdf]

VAF-based detection limit for *TP53_p.Arg282Trp*, *PTEN_p.Arg335Ter, IDH1_p.Arg132His*

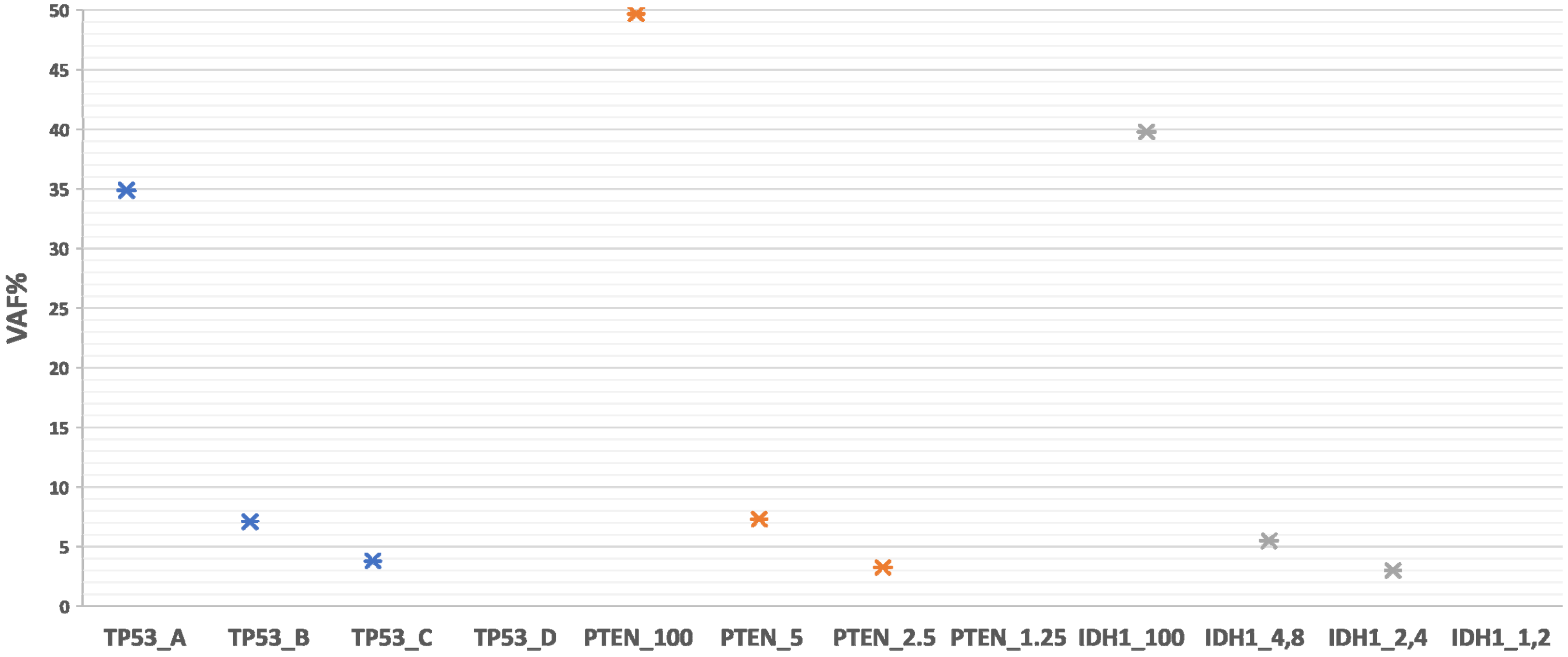

TP53_p.Arg282Trp	PTEN_p.Arg335Ter	IDH1_p.Arg132His
A: ID192 (100%)	A: ID192 (100%)	A: ID209 (100%)
B: ID192 (10%)	B: ID192 (10%)	B: ID209 (12%)
C: ID192 (5%)	C: ID192 (5%)	C: ID209 (6%)
D: ID192 (2,5%)	D: ID192 (2,5%)	D: ID209 (3%)

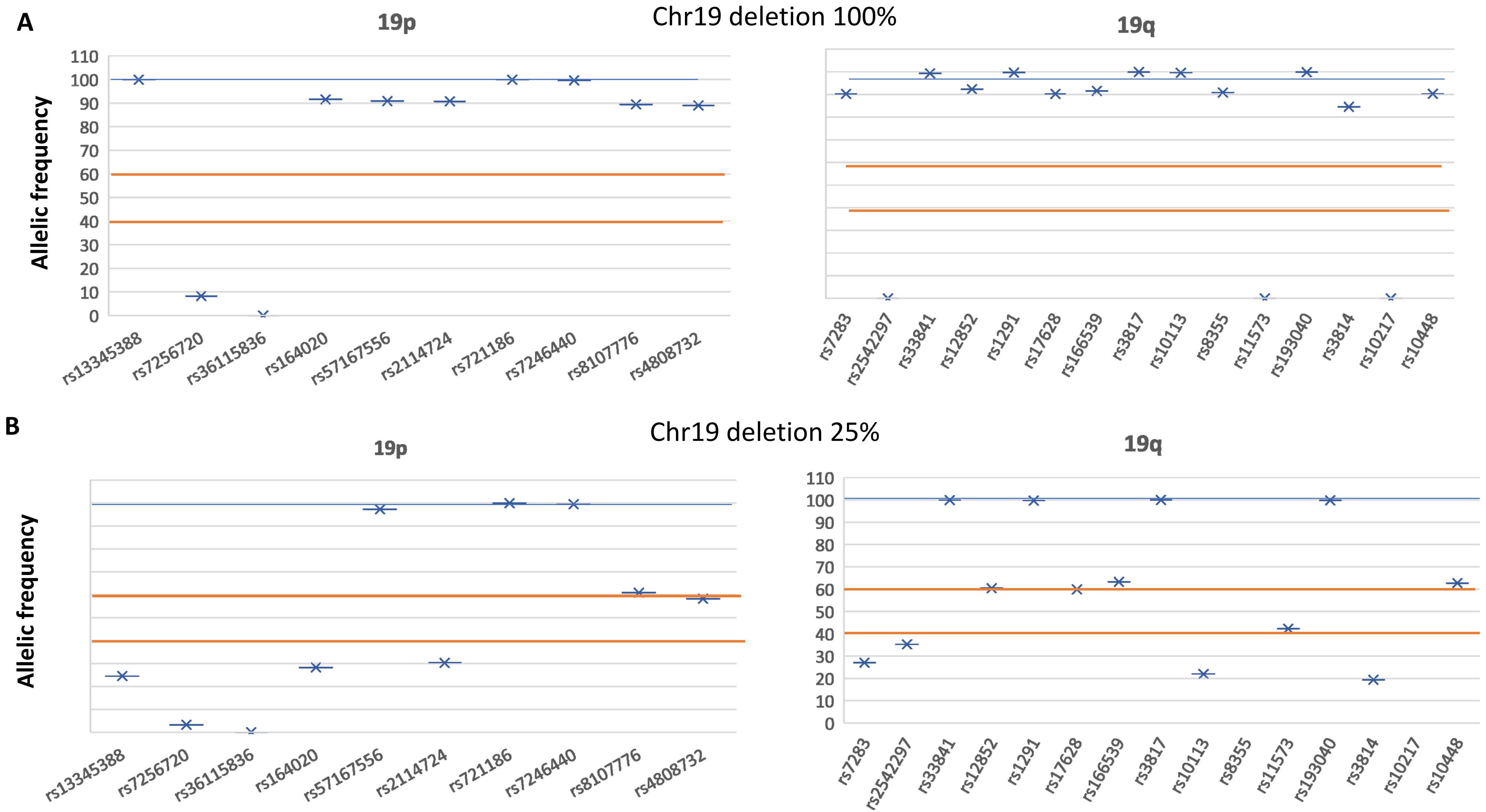

Calculation of the VAF-based detection limit from Chr9 deletion;A: undiluited reference DNA; B: reference Dna diluted in background of wild-Type Dna 1:25

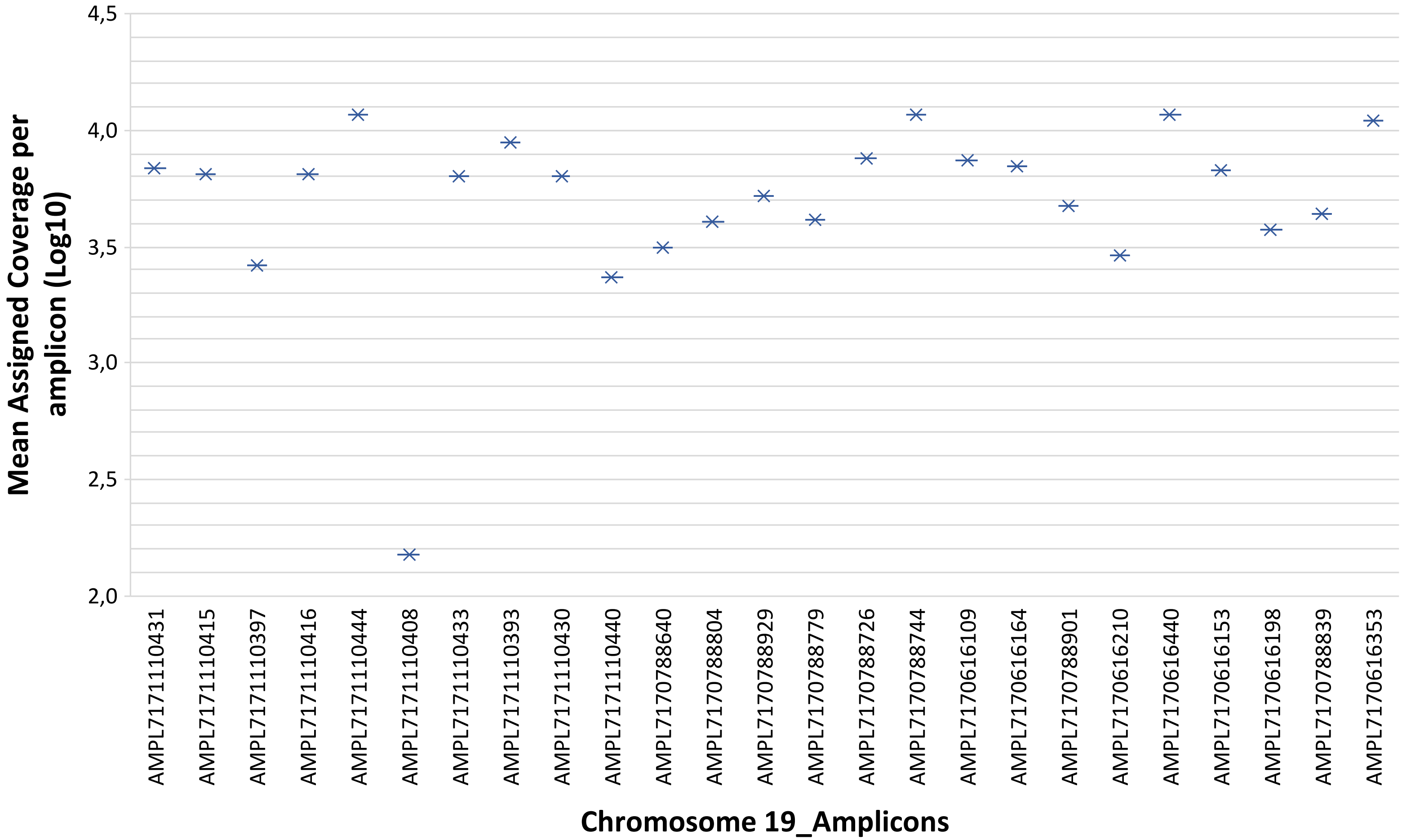

Figure S1. Amplicons' coverage of chr1. The graph shows the 29 amplicons used to assess copy number alterations of chr1.

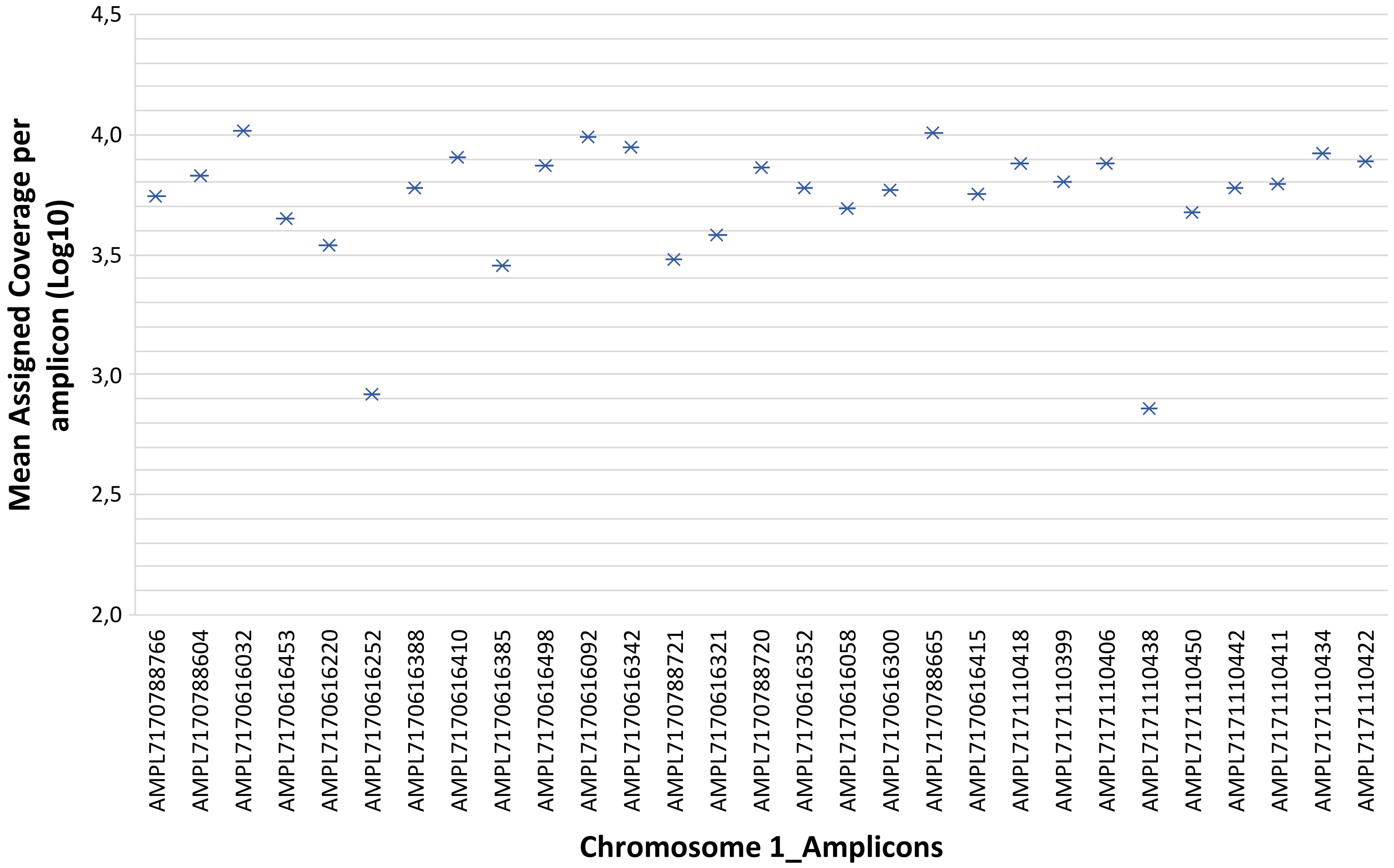

Figure S2. Amplicons' coverage of chr19. The graph shows the 25 amplicons used to assess copy number alterations of chr19.
